# Spine-pelvis index: a novel ratio to predict spinal growth in adolescent idiopathic scoliosis patients

**DOI:** 10.1186/1748-7161-10-S1-O17

**Published:** 2015-01-19

**Authors:** Hongda Bao, Zhen Liu, Zezhang Zhu, Feng Zhu, Wen Zhang, Xu Sun, Yong Qiu

**Affiliations:** 1 Spine Surgery, Drum Tower Hospital, Nanjing University Medical School, Nanjing, China

## Objective

To propose spine-pelvis index (SPI) as a novel ration in evaluating spinal growth.

## Background

Although the Bjure formula was often used to correct height loss caused by scoliosis, it only took account of the correction of major curve, ignoring the minor curves. Moreover, the growth of pelvis has not been well documented in literatures.

## Methods

A total of 75 female patients with AIS, 86 healthy age-matched girls and 40 age-matched patients with CS due to excrescent hemivertebra were included into this study. Long-cassette standing coronal radiographs of the spine and pelvis were obtained from the subjects in fist-on-clavicle position. The radiographic parameters were measured including length of spine (LOS), height of spine (HOS), length of thoracic vertebrae (LOT), height of thoracic vertebrae (HOT), width of pelvis (WOP), height of pelvis (HOP) and width of thorax (WOT). SPI was defined as the ratio between length of the spine and height of the spine, namely LOS/HOP. Four age cohorts (10~11 yrs, 12~13 yrs, 14~15 yrs and 16~18 yrs) were determined in AIS patients and normal controls. In addition, the theoretical value of LOS in CS patients was calculated according to the normal SPI measured in healthy controls.

## Results

HOS and HOT in AIS group were significantly lower than those in control group (P=0.041 and P=0.001), while LOS and LOT in AIS group were significantly larger (P=0.020 and P=0.027). SPI and LOT/HOP in AIS patients both showed significant increase from normal girls (P<0.05), implying abnormal growth pattern of spine relative to pelvis in AIS patients. In 12~13 yrs cohort and 14~15 yrs cohort, the downtrend of HOS/HOP and HOT/HOP was more obvious and LOT/LOS showed an uptrend in AIS patients, implying that growth velocity of lumbar vertebra was slower than that of thoracic vertebra. Based on the normal SPI with an average value of 2.256, the theoretical spinal length of the patients with CS was significantly smaller than the measured LOS in those patients (408.7mm vs. 424.5mm, P<0.05) and the difference value was similar between age groups (P>0.05), indicating that increased LOS was due to the excrescent hemivertebra.

## Conclusion

The current study demonstrated abnormal growth pattern of spine relative to pelvis in AIS patients. Since the HOP showed no difference between AIS and control groups, SPI could be used as a more reliable ratio to analyze the growth pattern in AIS patients and to predict the postoperative LOS in patient with CS.

